# Creating a knowledge translation trainee collaborative: from conceptualization to lessons learned in the first year

**DOI:** 10.1186/1748-5908-6-98

**Published:** 2011-08-25

**Authors:** Evelyn Cornelissen, Robin Urquhart, Vivian WY Chan, Ryan T DeForge, Heather L Colquhoun, Shannon Sibbald, Holly Witteman

**Affiliations:** 1 University of British Columbia - Okanagan, Faculty of Health and Social Development, 3333 University Way, Kelowna, B.C. V1V 1V7 Canada; 2 Dalhousie University, Interdisciplinary PhD Program, 1276 South Park Street; Room 804, Victoria Building; Halifax, N.S. B3H 2Y9 Canada; 3 University of British Columbia, Interdisciplinary Studies Graduate Program, Green College. Green Commons, Room 153A, 6201 Cecil Green Park Rd; Vancouver, B.C. V6T 1Z1 Canada; 4 The University of Western Ontario, Faculty of Health Sciences, 1151 Richmond St; London, O.N., N6A 5B9 Canada; 5 McMaster University, School of Rehabilitation Sciences, IAHS Building; 1400 Main St. W.; Hamilton, O.N., L8S 1C7 Canada; 6 University of Western Ontario, Faculties of Health Sciences and Information and Media Studies, 1151 Richmond St; London, O.N., N6A 5B9 Canada; 7 University of Michigan, Center for Bioethics and Social Science in Medicine, 300 North Ingalls Building 7C27, Ann Arbor, M.I. 48109-5429 USA

## Abstract

Trainees (*e.g*., graduate students, residents, fellows) are increasingly identifying knowledge translation as their research discipline. In Canada, a group of trainees have created a trainee-initiated and trainee-led national collaborative to provide a vehicle for trainees to examine the diversity of knowledge translation research and practice, and to link trainees from diverse geographical areas and disciplines. The aim of this paper is to describe our experience and lessons learned in creating the Knowledge Translation Trainee Collaborative. In this meeting report, we outline the process, challenges, and opportunities in planning and experiencing the collaborative's inaugural meeting as participant organizers, and present outcomes and learnings to date.

## Background

As the field of knowledge translation (KT) grows, trainees (*e.g*., graduate students, residents, fellows) are identifying KT as their research discipline. The most efficient way to advance the science of KT while developing new scholars will be to encourage new research collaborations and partnerships [1] and expose new researchers to multiple modes of inquiry and perspectives [2].

In 2008, KT Canada, along with its funding partners, established KT Summer Institutes (SI) to create opportunities for students involved in KT to learn from and connect with established KT researchers and other trainees [3]. These three- to four-day workshops have resulted in several subsequent collaborative efforts, including published papers [4,5] and successful grant applications [6,7]. Following the 2008 SI, several trainees identified a need for ongoing training and peer networking opportunities, and accordingly set out to create a collaborative that might serve to complement or supplement groups led and populated by experts in the field.

After an initial (unsuccessful) attempt to align a trainee networking meeting with a national health services research conference, two trainees [EC and VC] were awarded a grant [6], along with funding and in-kind support from other organizations, to hold an inaugural meeting to build such a collaborative. Thirteen additional SI participants [including RU, RD, HC, and HW] volunteered to plan the event. Following a formal application and review process supported by national KT experts, we held a two-day meeting in Winnipeg, Manitoba, 6 and 7 March 2010.

The aim of this paper is to describe our experience and lessons learned in creating the KT Trainee Collaborative (KTTC). We outline the process, challenges, and opportunities in planning and experiencing the KTTC inaugural meeting as participant organizers, and present outcomes and learnings to date.

### KTTC inaugural meeting

The inaugural meeting followed six months of planning via teleconference and email. The planning committee established three meeting objectives: forming our network; identifying areas for collaboration; and, with KT experts, reviewing potential gaps and training areas in KT research. We developed an agenda toward these goals and distributed a call for applicants.

Sixty-six applications were received from 14 Canadian universities. The applications were peer-reviewed by a group of planning committee members and KT experts. Review criteria included training, research interests, expectations, and anticipated contribution to the meeting. The aim of the review process was to assemble a group of trainees that could both contribute to and gain from capacity building in the field of KT. Thirty trainees attended the meeting: 11 from the planning committee and 19 accepted through the application process. Table 1 describes attendee characteristics.

**Table 1:** Attendee characteristics

	Planning Committee (n = 11) Count (percent) Number of institutions represented [institution names]	Successful Applicants (n = 19) Count (percent) Number of institutions represented [institution names]
Gender	1 male (9%) 10 female (91%)	5 male (26%) 14 female (74%)*

Province	1 Nova Scotia (9%) [Dalhousie University] 1 Prince Edward Island (9%) [University of Prince Edward Island] 4 Ontario (36%) 2 universities, 1 hospital [University of Western Ontario; McMaster University; St. Michael's Hospital] 1 Manitoba (9%) [University of Manitoba] 1 Alberta (9%) [University of Calgary] 2 British Columbia (18%) 1 university [University of British Columbia] 1 University of Michigan (9%)	1 Nova Scotia (5%) [Dalhousie University] 2 Quebec (11%) 2 universities [McGill University; Universite de Laval] 12 Ontario (63%) 6 universities [University of Waterloo; University of Western Ontario; McMaster University; University of Ottawa; York University; University of Toronto] 1 Manitoba (5%) [University of Manitoba] 2 Alberta (11%) 1 university [University of Alberta] 1 British Columbia (5%) [University of British Columbia]

Training	1 Post Doctoral/Fellows (9%) 9 Doctoral (82%) 1 Research Staff (9%)	5 Post Doctoral/Fellows (26%) 13 Doctoral (68%) 1 Master's (5%)

*The high percentage of female participants is representative of the proportion of applicants who were female.

The meeting involved four activities, interspersed by networking opportunities: setting the stage for future interaction/collaborations; goal-setting; KT expert-led discussion on potential gaps/training areas in KT research; and post-meeting planning. Dr. Ian Graham, Canadian Institutes of Health Research (CIHR) Vice President of KT, participated as the overall meeting facilitator.

#### Setting the stage

To begin the meeting, each attendee introduced her/himself in a two-minute prepared talk describing her/his research, career stage, KT interests, and something about her/himself. These introductions oriented us to each other's work, provided opportunity to practice a fundamental skill in scholarship (concisely describing one's work to an interdisciplinary audience), and ensured that all attendees participated early on.

Two planning committee members [RD, HC] then facilitated a 'creating space' exercise to establish principles for interaction and a sense of 'safety.' We acknowledged that over time our 'space' would likely take different forms because it was expected that, going forward, the KTTC would meet both in-person and virtually. However, the focus of this exercise was to co-construct shared principles for relating to and with one another in the context of this meeting. We also acknowledged that all shared spaces are inherently characterized by competing interests, agendas, needs, and experiences. Our aim was to encourage these interests and to conceive of new ways of working together wherein we each feel included and valued. The facilitators invited small groups to consider questions about creating an inclusive and engaging workspace, then collated responses to form a foundation of shared understandings upon which we continued our meeting.

#### Goal setting

To make sense of our group's goals and expectations, first, each attendee identified his/her individual goals. Then, we iteratively and thematically categorized individual goals into four collective goals: networking, collaborative learning, collaborative work, and professional development. Although it was evident that attendees held diverse research interests in a broad range of health areas, the four collective goals reflected our common perspectives and desires.

#### Expert-led discussions

KT experts presented their perspectives and experiences in three different areas identified as representing fruitful areas for further research and training: behavioural theory in policy/decision support (Dr. Jamie Brehaut [8]), unintended consequences of KT (Dr. Maria Mathews [9]), and critical/qualitative inquiry in KT (Dr. Annette J. Browne [10]). The experts then facilitated small group discussions based on their presentation topics.

#### Post-meeting planning

We ended the meeting by developing a post-meeting plan. We brainstormed activities to help us meet our collective goals, and elected a Steering Committee to oversee activities and establish a governance structure. We also reached agreement on preferred technological mechanisms to support ongoing collaboration with limited resources.

#### Meeting evaluation

Using a 'two stars and a wish' exercise to garner feedback, wherein attendees were asked to list two things about the meeting they enjoyed and one thing they would like to see changed or improved, attendees indicated they felt: encouraged to be engaged in the process ('an interactive, open, and respectful atmosphere'); accomplished ('amazing how much we accomplished in 1.5 days'); and well-facilitated ('gradual process'; 'inductive approach'). Members wished for: a clearer vision ('more concrete sense of how to collaborate immediately') and more opportunities to network ('formal and informal time').

### KTTC outcomes

From this meeting, the KTTC emerged as a uniquely trainee-initiated and trainee-led national collaborative to provide a vehicle for trainees to examine the diversity of KT research and practice, and to link trainees from diverse geographical areas and disciplines. We identify as a type of community of practice (CoP) [11] for KT trainees in Canada, with the underlying belief that junior researchers and practitioners can acquire valuable KT knowledge and skills, and engage in beneficial collaborative learning and working processes, through social relationships with their peers. A recent systematic review [12] identified four characteristics of CoP groups that we perceive are shared by the KTTC: members interact with one another in formal and informal settings; members share knowledge with one another; members collaborate with one another to create new knowledge; and groups promote the development of a shared, professional identity amongst members.

To date, we have created a formal collaborative, and opened to new membership in February 2011. We have identity, mission, and vision statements (Table 2), a governance structure to guide our continued growth, and working groups to develop and implement specific activities toward our collective goals [13].

**Table 2:** Identity, mission, and vision statements

Who We Are	The Knowledge Translation Trainee Collaborative (KTTC) is a community of practice in knowledge translation (KT). Members of the KTTC are junior researchers and practitioners who are interested in continuing to learn about and advance the field of KT, and who want to collaborate and build networks with other KT trainees. We define trainees as students, graduate students, postdoctoral fellows, faculty, community learners, scientists/researchers from a wide spectrum of academia, healthcare professionals, healthcare administrators, and/or others who are new to KT and are interested in actively exploring and developing KT research and practice.
**Vision**	We envision a sustainable network that provides accessible, ongoing opportunities for collaboration and learning; represents diversity of thought in KT theory, methods and tools; and grows and advances the field of KT.

**Mission**	We are creating our vision by sharing opportunities for: 1. collaborative learning 2. collaborative work 3. building networks 4. career development

We have assembled a database of current and potential collaborators, held a second meeting in May 2010 to coincide with a national conference [14] and a third meeting in April 2011 funded by another peer-reviewed grant [7], and launched a group blog hosted on a national KT website [15]. Traditional academic outputs include poster presentations at two academic conferences [16,17] and a successful funding application for a follow-up meeting [7]. That trainees from this collaborative have received two peer-reviewed grants from a national health research funding agency without a traditional independent investigator is a significant achievement that speaks to the commitment and initiative of members.

### Moving forward

As the meeting concluded, a number of challenges and opportunities were identified (Table 3). In order to respond to these challenges and opportunities, and particularly in order to maintain our momentum, attendees decided to devote the subsequent six to twelve months to creating a vision, governance structure, and technological infrastructure before opening the KTTC to new membership. As we plan the KTTC's future, we are challenged by our status as trainees and concerns about sustainability. Our commitment to the collaborative requires creative thinking, and belief that time invested now--in our group, career development, and collaborative learning/work--will yield future benefit. Such challenges are common in the early stages of many social innovations that eventually succeed [18], and challenges often coexist with opportunities.

**Table 3:** Challenges and opportunities identified during and after the inaugural meeting

Challenges	Opportunities
▪ Ensuring the KTTC achieves diversity (*e.g*., in research methodologies, disciplines, geography, and academic vs. practice environments) ▪ Maintaining momentum ▪ Ensuring active and effective external and internal communication ▪ Providing value for all members (*e.g*., senior and junior) ▪ Meeting the expectations of all active members ▪ Workload for members involved in administration	▪ Growing academic and administrative interest in KT ▪ Increasing number of KT trainees across Canada* ▪ Committed membership comprised of trainees from diverse backgrounds and experiences ▪ Members who value innovative thinking on and approaches to KT ▪ A 'safer' space for trainee dialogue and discourse ▪ Ability to complement and network with existing KT research groups and agencies

*The increasing number of KT trainees is reflected by the growing number of trainee awards and opportunities in Canada: *e.g*., the Canadian Institutes of Health Research (CIHR) now provides doctoral research awards and new investigator awards focused on KT science and KT Canada holds a Strategic Training Initiative in Health Research grant from CIHR to create an internationally-recognized national training initiative to train graduate and post-doctoral students in KT and KT science.

### Key Learnings

Three key learnings that may be applicable to other groups emerged from the meeting.

First, we found the term 'trainee' was more contentious than we had foreseen. Some members felt the term would soon cease to apply as members began their careers. Others, however, saw the word as one that connotes life-long learning and as applicable to KT learners (within or outside academe) at all career stages. Eventually, the group adopted the latter interpretation and clarified it in our Mission and Vision statements.

Second, as with any large group aiming for inclusivity of all members, we encountered tension between the desire for a 'flat' structure--with its potential for time-consuming and stagnating discussions--and the inevitable hierarchy of a formal collaborative. Further, at the meeting, open-ended questions to attendees such as, 'How would you like to do this?' were sometimes more off-putting than welcomed. Ultimately, the planning committee reconciled these issues by being transparent about the 'tyranny of structurelessness' [19] and by inviting all attendees to collectively endorse some executive decision-making and organizational structure, grounded in our principles from the creating space activity. Such an endorsement later empowered the Steering Committee to make decisions required to build the collaborative.

Third, in the same way that facilitation is important in KT initiatives [20,21], we found it equally crucial in the development of our collaborative. While the continua that Harvey *et al*. [21] present to conceptualize facilitation might be interpreted as an either/or dichotomy (*i.e*., either task-oriented, doing for others, or holistic/enabling others), our experience was one of and/both. As facilitators, we needed to provide some structure to the meeting, but also needed to ensure that each activity unfolded in a way that left all attendees feeling their contributions were authentically valued. In other words, and as Harvey *et al*. [21] postulate, effective facilitators need to be flexible and possess a range of task-focused and enabling skills that can be employed according to contextual needs. We relied on tenets of empowering dialogue [22] by focusing on attendees' concerns, using (inter)active learning strategies, and engaging attendees in processes to identify their needs and priorities. The integration of task and holistic facilitation, with empowering dialogue, helped us realize our meeting objectives. Beyond enabling group formation, it is our sense that strategies such as these also serve to identify and respect unique disciplinary perspectives of group members, which is essential in fostering a KT climate that espouses growth from multi- to inter-disciplinarity [23]. In this way, we feel the KTTC also serves to start addressing the challenges of cross-disciplinary collaboration.

## Conclusion

Advances in KT will necessitate multiple perspectives, research approaches [2], and open cross-pollination amongst disciplines [24]. By bringing together a diverse group of trainees, the trainee-led KTTC offers the potential to complement or supplement the formal training activities of expert- or mentor-led groups, such as KT Canada, by allowing for more peer interaction and peer leadership opportunities. Through such experiences, we expect members of the collaborative to better achieve the benefits of inter- and trans-disciplinarity [23]; that is, we expect the field and our own individual research products to be improved through exposure to diverse and challenging ideas in a community of KT scholar and practitioner peers. Expanded membership and continued commitment from KT trainees will, we hope, produce further collaborative learning and work experiences that can continue to contribute to the field of KT and benefit other trainees in the field.

To learn more about membership in the KTTC, go to http://www.ktclearinghouse.ca/kttc/.

## Competing interests

The authors declare that they have no competing interests.

## Authors' contributions

EC developed the outline of the paper, coordinated the content from all authors, and prepared the first draft with assistance from RU and HW. All authors contributed to the final content. EC, RU, VC, and HW edited the final draft. EC, RD, HW, RU, and HC revised the manuscript according to reviewers' comments. All authors have read and approved the final manuscript.
